# Molecular and clinical characterization of PTPN2 expression from RNA-seq data of 996 brain gliomas

**DOI:** 10.1186/s12974-018-1187-4

**Published:** 2018-05-15

**Authors:** Peng-fei Wang, Hong-qing Cai, Chuan-bao Zhang, Yan-Michael Li, Xiang Liu, Jing-hai Wan, Tao Jiang, Shou-wei Li, Chang-Xiang Yan

**Affiliations:** 1 Department of Neurosurgery, Sanbo Brain Hospital, Capital Medical University, Building 1, Ward 6, Xiang Shan Yi Ke Song Road 50, Haidian, Beijing, China; 2 Department of Neurosurgery, National Cancer Center/Cancer Hospital, Chinese Academy of Medical Sciences and Peking Union Medical College, Beijing, China; 3 Department of Neurosurgery, Beijing Tiantan Hospital, Capital Medical University, Beijing, China; 4 Beijing Neurosurgical Institute, Beijing, China; 5 Chinese Glioma Genome Atlas Network (CGGA), Beijing, China; 6 Department of Neurosurgery and Oncology, University of Rochester Medical Center, Rochester, NY USA; 7 Department of Imaging Sciences, University of Rochester Medical Center, Rochester, NY USA

**Keywords:** Glioma, Immune response, Immune cells, PD-1, PTPN2

## Abstract

**Background:**

Immune checkpoint inhibitors have been shown to promote antitumor immunity and achieve durable tumor remissions. However, certain tumors are refractory to current immunotherapy. These negative results encouraged us to uncover other therapeutic targets and strategies. PTPN2 (protein tyrosine phosphatase, non-receptor type 2) has been newly identified as an immunotherapy target. Loss of PTPN2 sensitizes the tumor to immunotherapy via IFNγ signaling.

**Methods:**

Here, we investigated the relationship between PTPN2 mRNA levels and clinical characteristics in gliomas. RNA-seq data of a cohort of 325 patients with glioma were available from the Chinese Glioma Genome Atlas and 671 from The Cancer Genome Atlas. R language, GraphPad Prism 5, and SPSS 22.0 were used to analyze data and draw figures.

**Results:**

PTPN2 transcript levels increased significantly with higher grades of glioma and in isocitrate dehydrogenase (IDH) wild-type and mesenchymal subtype gliomas. A comprehensive biological analysis was conducted, which indicated a crucial role of PTPN2 in the immune and inflammation responses in gliomas. Specifically, PTPN2 was positively associated with HCK, LCK, MHC II, and STAT1 but negatively related to IgG and interferon. Moreover, canonical correlation analysis showed a positive correlation of PTPN2 with infiltrating immune cells, such as macrophages, neutrophils, and CD8^+^ T cells. Clinically, higher levels of PTPN2 were associated with a worse overall survival both in patients with gliomas and glioblastomas.

**Conclusion:**

PTPN2 expression level was increased in glioblastomas and associated with gliomas of the IDH wild-type and mesenchymal subtype. There was a close correlation between PTPN2 and the immune response and inflammatory activity in gliomas. Our results show that PTPN2 is a promising immunotherapy target and may provide additional treatment strategies.

**Electronic supplementary material:**

The online version of this article (10.1186/s12974-018-1187-4) contains supplementary material, which is available to authorized users.

## Background

Gliomas are the most common malignant brain tumors, accounting for 74.6% of all malignant central nervous system (CNS) tumors [1]. Despite the progress of multimodal conventional therapies, there is limited improvement in the overall survival (OS) of patients with gliomas [2]. Immune checkpoint inhibitors (ICIs) have achieved great success in the treatment of solid tumors, including melanoma [3], non-small cell lung cancer [4], and renal cell carcinoma [5]. Moreover, the discovery of lymphatic vessels in the CNS raised the hope of achieving immunotherapy in brain tumors [6]. However, there is a low objective response of current immunotherapies for most gliomas.

The combination of two different ICIs results in better clinical outcomes in patients with melanomas than does ICI alone [7, 8]. In previous studies, PD-L1 (programmed death-ligand 1) [9], TIM3 (T-cell immunoglobulin mucin-3) [10], and IDO1 (indoleamine 2,3 dioxygenase 1) [11] transcript levels were strongly correlated with immune responses and prognosis in gliomas. Experimental results have also shown that the combination of anti-PD-1 and anti-TIM3 therapies could achieve a longer OS than anti-TIM3 alone [12]. These exciting results prompted us to uncover other immune targets in gliomas.

PTPN2 (protein tyrosine phosphatase, non-receptor type 2) has been recently identified as a novel cancer immunotherapy target. PTPN2 is frequently mutated and repressed in hematological malignancies, negatively regulating JAK/STAT signaling [13]. Loss of PTPN2 results in increased numbers and activation of CD8^+^ T cells, promoting an autoimmune syndrome [14] and enhances interferon γ (IFN-γ) and interleukin-6 (IL-6) secretion in vitro [15, 16]. Loss of PTPN2 also sensitized tumors to immunotherapy in models of melanoma and colon cancers. Furthermore, PTPN2 transcript levels are upregulated in human cancers that are refractory to current immunotherapy [17]. Unfortunately, there is no comprehensive report on PTPN2 in gliomas. Therefore, we utilized the Chinese Glioma Genome Atlas (CGGA) dataset, which consists of 325 glioma samples, to investigate PTPN2 in gliomas. Moreover, The Cancer Genome Atlas dataset (TCGA; http://cancergenome.nih.gov/) was used as a validation group. To the best of our knowledge, this is the first study to molecularly and clinically characterize PTPN2 expression in gliomas.

## Methods

### Patients and data collection

The data, including clinicopathological characteristics and whole genome RNA-seq data of 325 patients with glioma (World Health Organization (WHO) grades II–IV) from the CGGA dataset, have been described in our previous study [10]. Besides, data from TCGA were downloaded from the cBioPortal (http://www.cbioportal.org/), and 671 gliomas (WHO grades II–IV) were analyzed in this study.

### Related signature identification

All RNAseq data were log-transformed. Significantly related genes with PTPN2 expression were retrieved by using the Pearson correlation analysis. Gene ontology (GO) analyzed related gene sets and relevant biological functions on the DAVID website (http://david.ncifcrf.gov/) [18]. Cluster 3.0 (Human Genome Center, Institute of Medical Science, University of Tokyo, Japan) was used to plot heat maps of PTPN2 expression and correlated genes involved in inflammatory or immune responses. In addition, the gene set variation analysis (GSVA) package of R language was used to transform gene expression measurements of each sample into scores for inflammatory response metagenes.

### Statistical analysis

The discrepancies in PTPN2 expression between grade, isocitrate dehydrogenase (IDH) mutation status, and expression subtype were evaluated by using the Student *t* test. R language was used for Pearson correlation and correlogram analysis. The correlation between PTPN2 expression and different inflammatory cell types was examined by canonical correlation using the SPSS 22.0 software. When investigating the prognostic value of PTPN2, the Cutoff Finder was used to determine the optimal cutoff point of PTPN2 transcript level [19]. Besides, log-rank and Cox regression analysis were applied to investigate the prognostic value of PTPN2 using GraphPad Prism 5 and SPSS 22.0, respectively. All statistical tests were two-sided. A *p* value lower than 0.05 was considered statistically significant.

## Results

### PTPN2 transcript levels in glioma with different grades and IDH mutation status

**Fig. 1:**
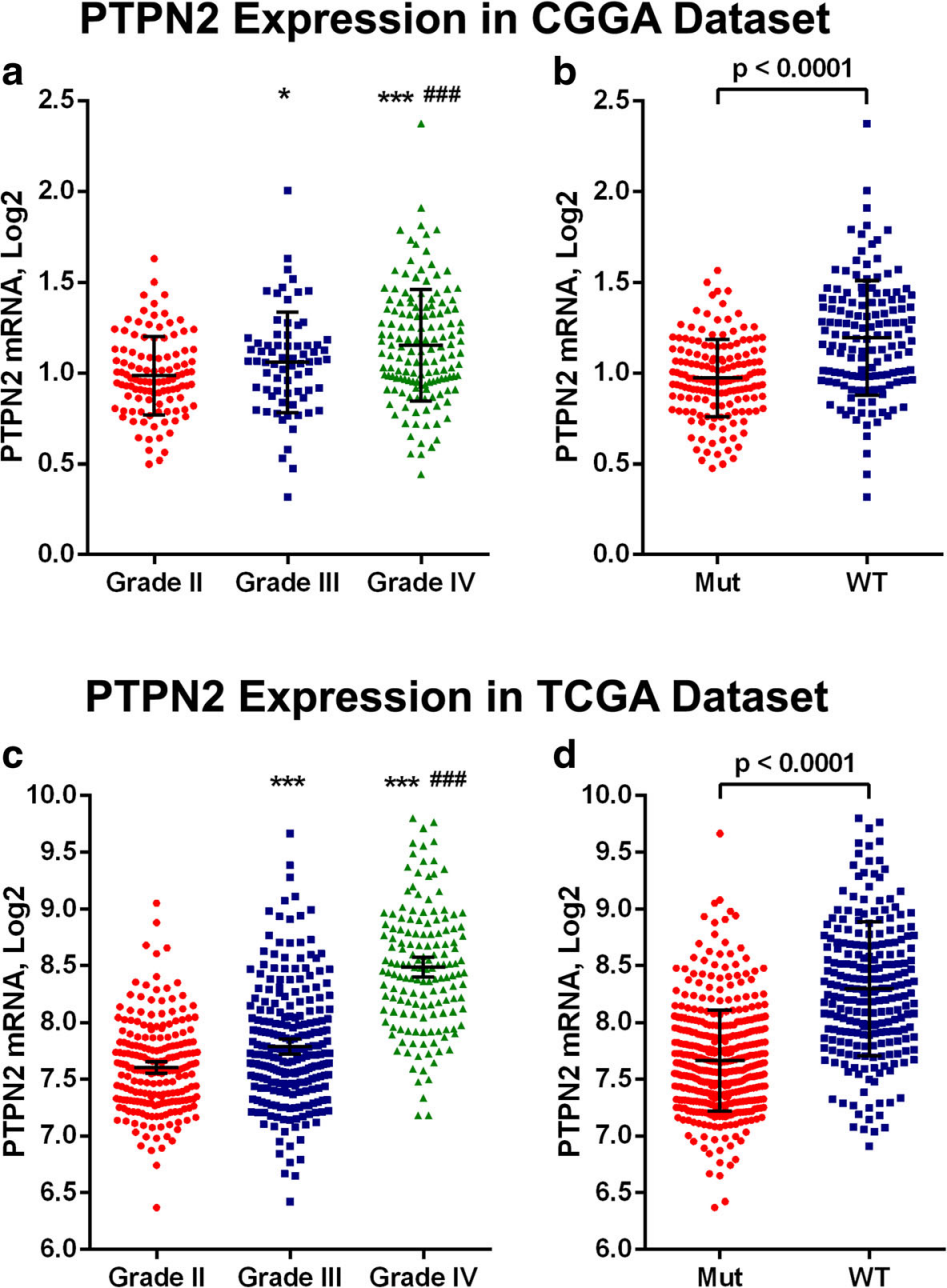
PTPN2 transcript levels increase with tumor grades and in IDH-WT gliomas. **a** In the CGGA dataset, mRNA levels of PTPN2 were compared between grade II (*n* = 109) and grade III (*n* = 72) gliomas, grade IV glioblastoma (GBM; *n* = 144), and **b** in IDH-mutant (Mut; *n* = 158) and IDH wild-type (WT; *n* = 142) gliomas. **c** In the TCGA dataset, PTPN2 transcript levels were compared between grade II (*n* = 216) and grade III (*n* = 241) gliomas, grade IV glioblastoma (GBM; *n* = 155), and **d** IDH-Mut (*n* = 429) and IDH-WT (*n* = 234) gliomas

The RNA-seq data of glioma from CGGA and TCGA databases were extracted to analyze the expression pattern of PTPN2 in gliomas. We found that PTPN2 transcript levels increased with the tumor grade. In the CGGA cohort, glioblastoma (GBM) showed higher levels of PTPN2 than grade II and grade III gliomas (Student’s *t* test, *p* < 0.001 and 0.030, respectively; Fig. 1a). However, the difference in PTPN2 levels between grade II and grade III gliomas was modest in the CGGA cohort (*p* = 0.0497, Fig. 1a). It is well established that IDH mutations significantly distinguish gliomas in terms of genetic changes and survival [20]. Moreover, the 2016 WHO incorporated a classification of gliomas into grades II–III and GBM on the basis of IDH mutation status [21]. We also analyzed PTPN2 expression on the basis of IDH mutation status. The transcript levels of PTPN2 were significantly elevated in wild-type IDH grade II–III gliomas (Student’s *t* test, *p* = 0.003; Fig. 1b). All these results were validated in the TCGA cohort (Fig. 1c, d). Furthermore, we compared PTPN2 transcript levels with the O6-methylguanine DNA methyltransferase (MGMT) status. The results show that the transcript levels of PTPN2 were decreased in gliomas with MGMT promoter methylation in both CGGA (*p* = 0.013, Additional file 1: Figure S1A) and TCGA (*p* < 0.001, Additional file 1: Figure S1B) datasets.

### PTPN2 expression is upregulated in the mesenchymal molecular subtype

**Fig. 2:**
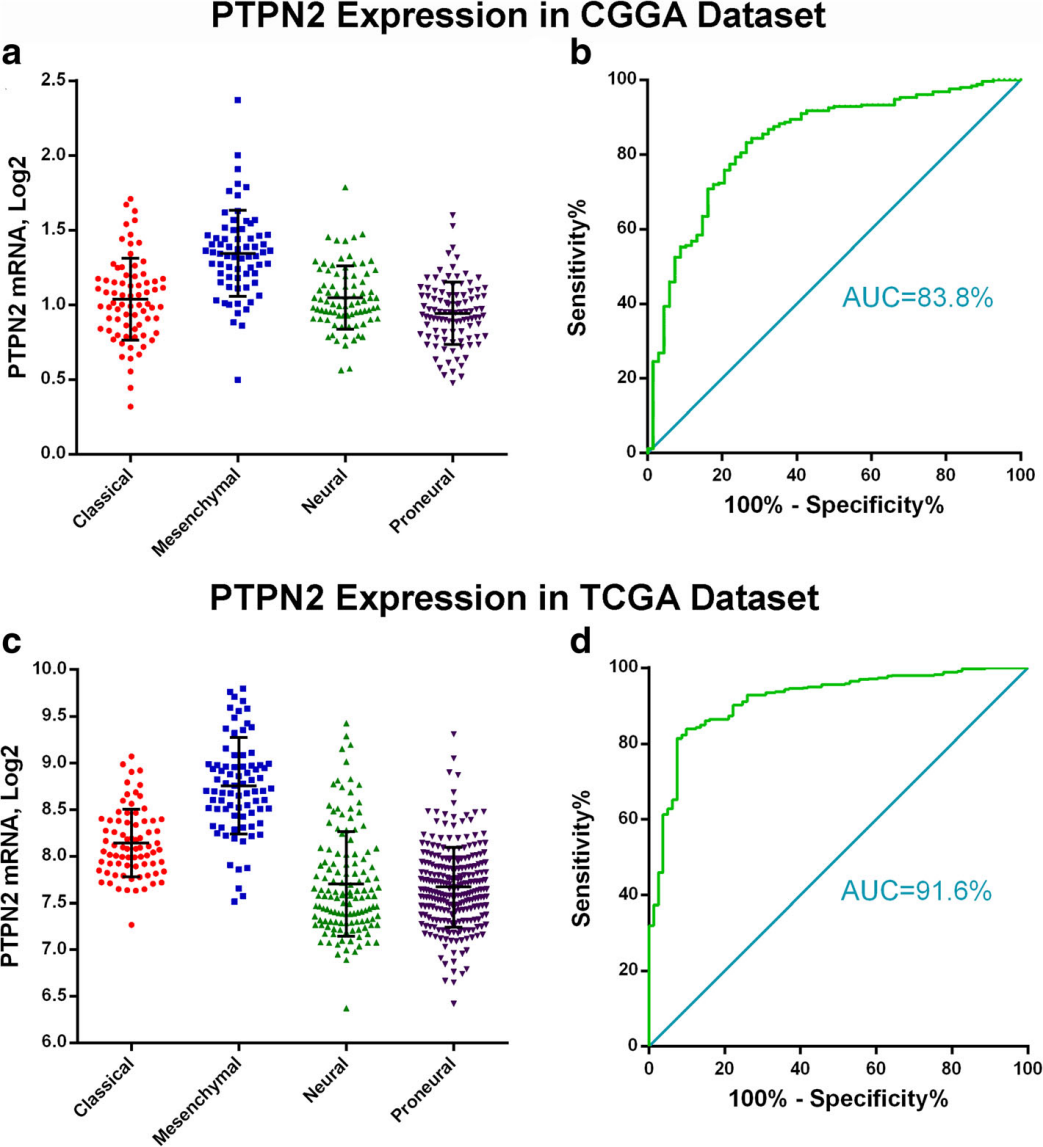
PTPN2 mRNA level is highly increased in the mesenchymal molecular subtype of glioma (**a** and **c**). ROC curve analysis reveals the predictive value of PTPN2 in the mesenchymal subtype (**b** and **d**)

Given the most advocated gene expression-based classification [22], we investigated the transcript levels of PTPN2 according to the transcriptional subtypes. We found that PTPN2 expression was significantly higher in the mesenchymal subtype than in the other subtypes of the CGGA cohort (Fig. 2a). It was also validated in the TCGA cohort (Fig. 2c). Next, the ROC curves for PTPN2 and mesenchymal subtype were plotted. The area under the curve (AUC) was 83.8% in the CGGA cohort and 91.6% in the TCGA cohort (Fig. 2b, d).

### PTPN2-related biological signatures

**Fig. 3:**
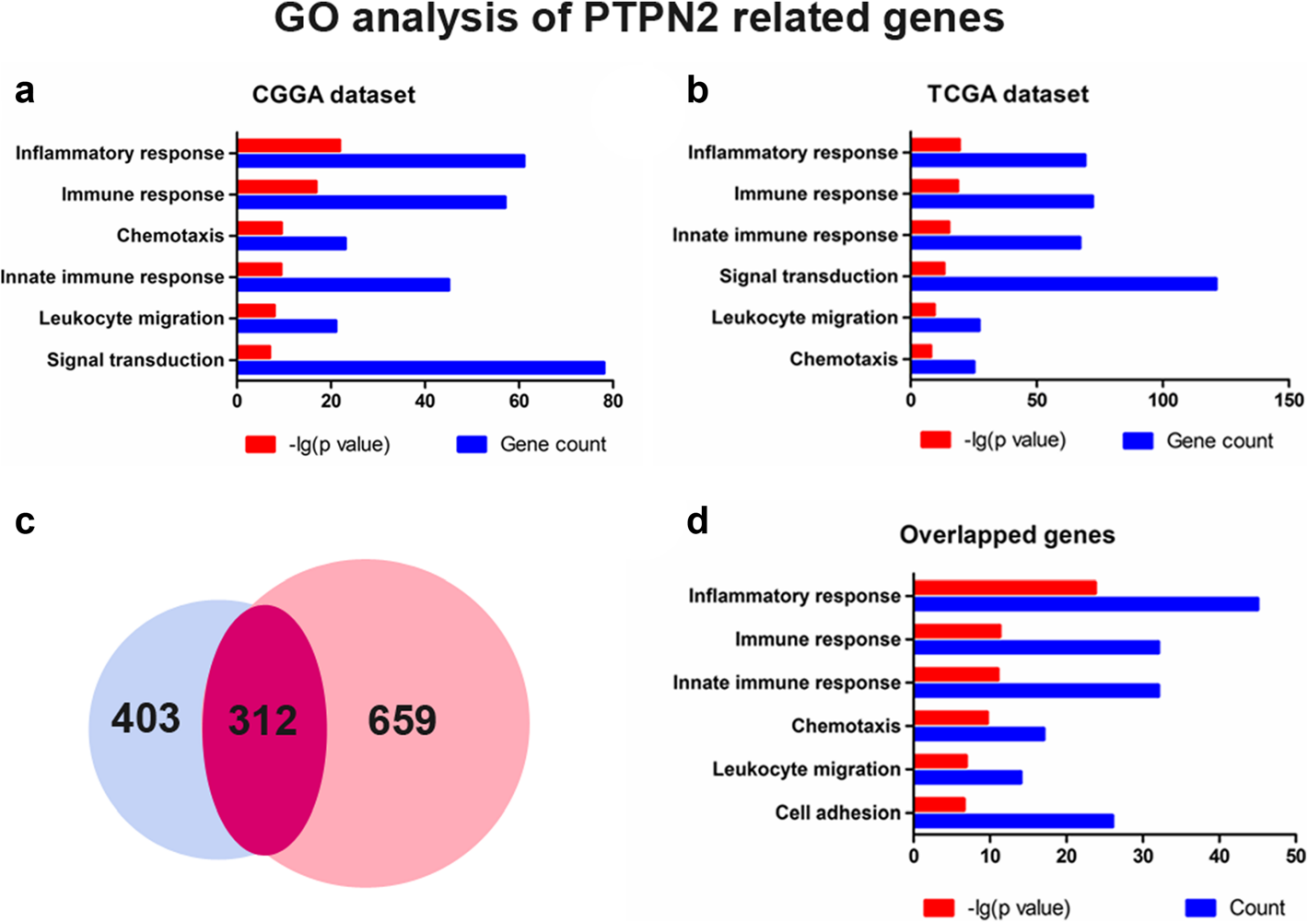
PTPN2 is strongly related to immune functions in glioma. **a**, **b** GO analysis of the CGGA and TCGA datasets shows that PTPN2 is involved in the immune response, inflammatory response, and other GO immune functions. **c** Venny mapping shows 312 genes that are overlapping. **d** GO analysis of 312 overlapping genes, showing a more reliable relationship between PTPN2 and immune functions

To explore the main biological signatures of PTPN2 in glioma, we investigated the related genes by the Pearson correlation analysis (|*r*| > 0.6 and *p* < 0.05). In total, 715 and 971 genes were selected as significantly correlated with PTPN2 expression in the CGGA and TCGA datasets, respectively. We then analyzed the biological function of related gene sets by GO analysis on the DAVID website. The results show that related genes from the two datasets were mainly involved in inflammatory response and immune response, as well as in chemotaxis, leukocyte migration, and innate immune response (Fig. 3a, b). We further investigated the function of 312 genes that overlapped the two gene sets using GO analysis and found similar results, which suggests a more reliable relationship between PTPN2 and inflammatory response/immune response in glioma (Fig. 3c, d). Additional file 2: Table S6 shows a list of the 20 genes whose expression was most significantly positively and negatively correlated with PTPN2 expression.

### PTPN2-related immune response

**Fig. 4:**
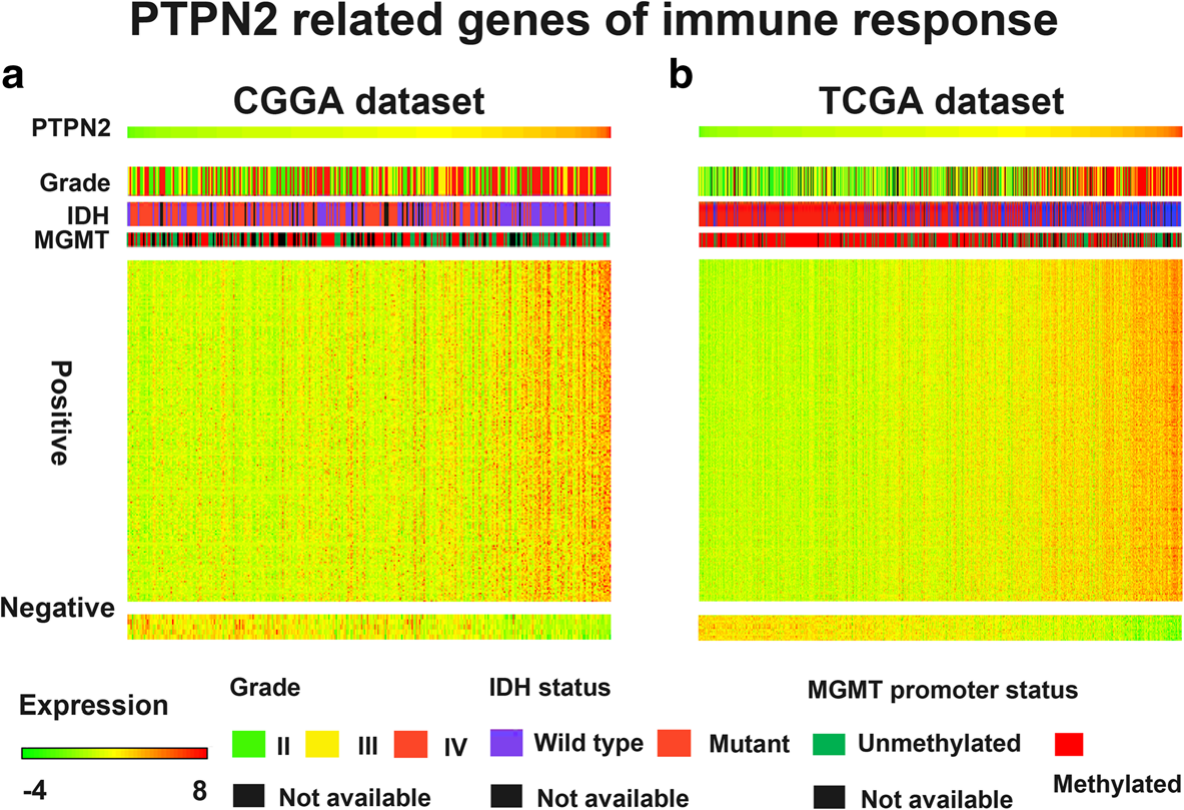
**a**, **b** PTPN2-related immune response in the CGGA and TCGA databases. A heat map displays the clinicopathological parameters, PTPN2 expression, and most associated gene expression in the immune response of gliomas from the CGGA and TCGA datasets

We downloaded gene sets related to every immune system process that functions in the calibrated response of an organism to an internal or invasive threat from the AmiGO 2 website (http://amigo.geneontology.org/amigo). A total of 2112 genes were found to be involved in immune responses in humans (Additional file 3: Table S1). We then matched these 2112 genes with PTPN2 significantly related genes in the two databases. In total, 229 positively and 6 negatively correlated immune genes in CGGA and 291 positively and 16 negatively correlated immune genes in TCGA dataset were defined. A detailed list of these genes is shown in Additional file 4: Table S2. All these genes from both datasets were selected for heat map analysis (Fig. 4a, b). Similar to previous studies [17, 23], PTPN2 expression was strongly linked with immune responses in glioma. Expression of genes involved in the immune response was positively related to glioma grades (Additional file 5: Figure S2A, B) but negatively associated with IDH1 mutation (Additional file 5: Figure S2C, D) and MGMT promoter methylation (Additional file 5: Figure S2E, F).

### PTPN2-related inflammatory response

**Fig. 5:**
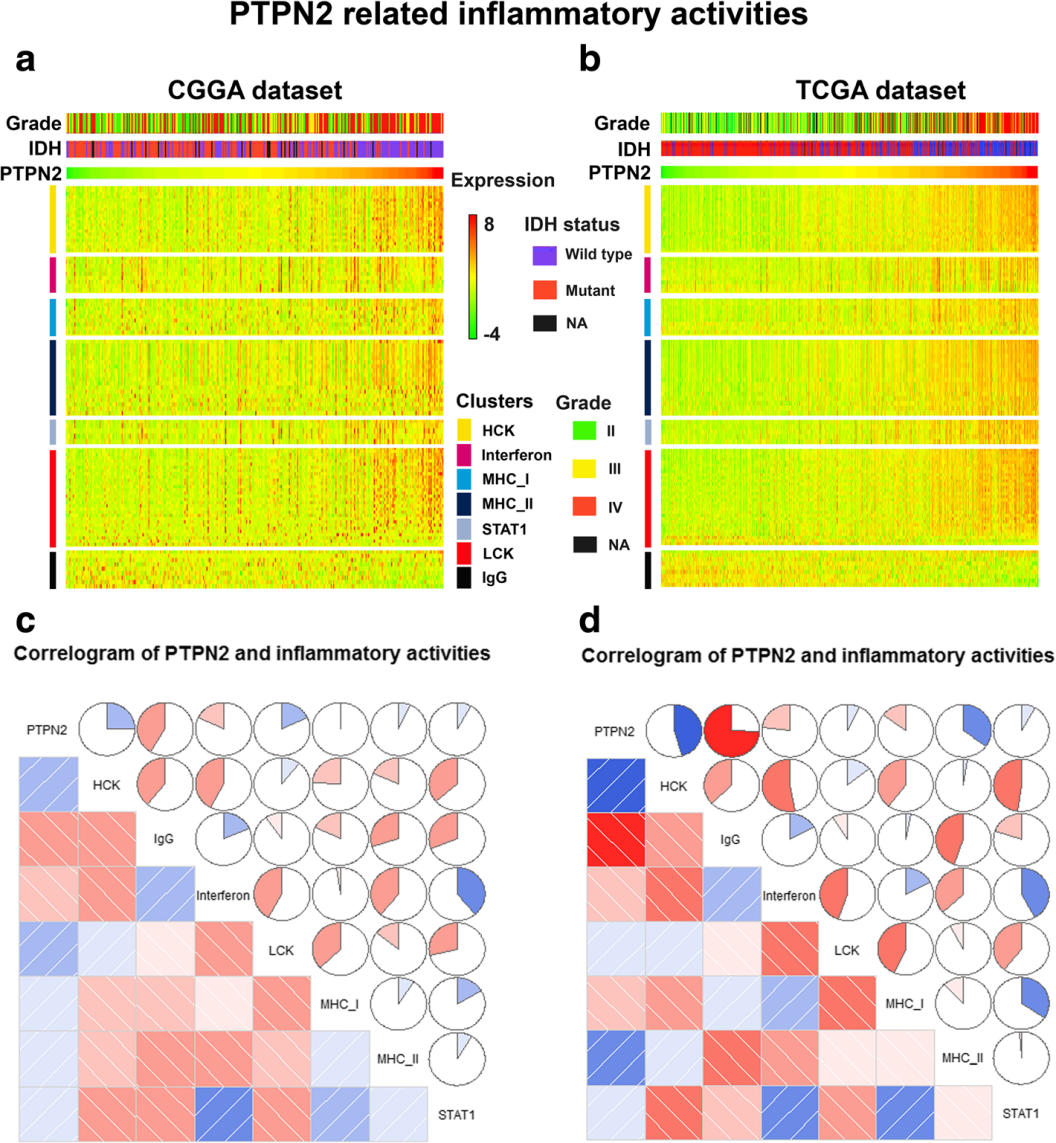
PTPN2-related inflammatory response. **a**, **b** Heat maps displaying the clinicopathological parameters, PTPN2 expression, and seven related metagenes from the CGGA and TCGA datasets. As shown, PTPN2 expression positively correlates with HCK, LCK, MHC II, and STAT1 gene expression but is negatively related to IgG. **c**, **d** Correlograms were established based on the relationship between PTPN2 expression and GSVA enrichment scores for these seven metagenes. PTPN2 expression is positively correlated with HCK, LCK, MHC II, and STAT1 in the CGGA and TCGA cohorts but negatively correlated with IgG and interferon. The circles were filled in blue clockwise for positive values and in red anticlockwise for negative values. The intensity of color increases with the correlation value moving away from 0

To better understand PTPN2-related inflammatory activities, we used seven well-established metagenes [24] (comprising 104 genes in total) that act as surrogate markers of different immune cell types (Additional file 6: Table S3). Glioma grade, IDH status, PTPN2 expression, and metagenes of patients were visualized as heat maps. As shown in Fig. 5a, b, PTPN2 expression was positively correlated with HCK, LCK, MHC II, and STAT1 in the CGGA and TCGA cohorts but negatively with IgG, a marker for B cells. To verify the heat map analysis, we used GSVA to transform gene expression measurements into enrichment scores for these metagenes. The Pearson correlation analysis was measured between PTPN2 expression and scores of the seven metagenes. Correlograms were generated by R language, which were similar to the abovementioned results. Furthermore, GSVA confirmed that the PTPN2 transcript level was negatively associated with IgG and interferon levels (Fig. 5c, d).

### Relationship between PTPN2 and infiltrating immune cells

**Fig. 6:**
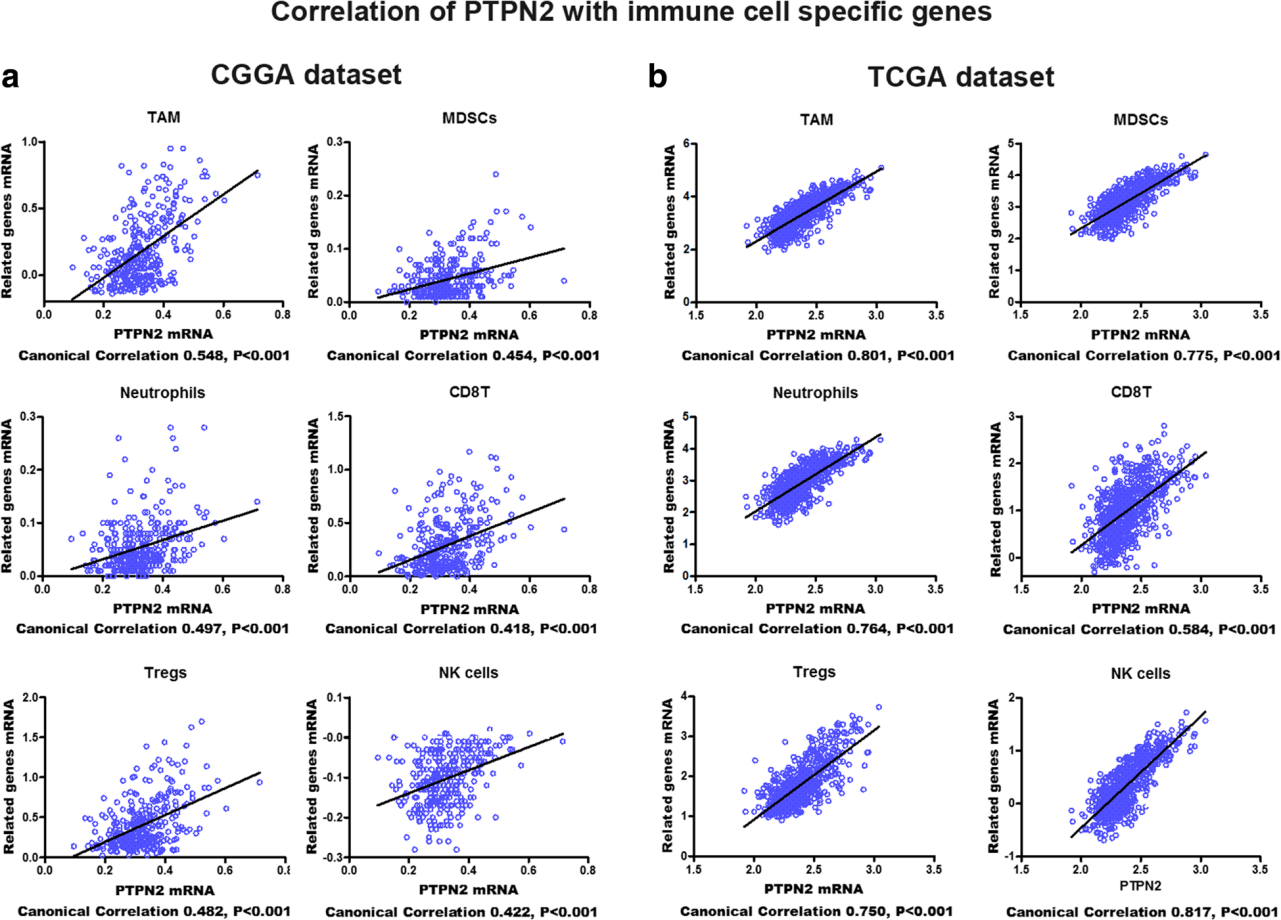
**a**, **b** Correlation of PTPN2 mRNA with immune cell-specific marker genes. Each open circle represents a single patient with glioma. A regression line was fitted to the dot plot

Immune cells are crucial effector cells in immune and inflammatory responses. In glioma, several immune cell types infiltrating tumor specimens were found to have a biological significance in the regulation of cancer progression and prognosis [25, 26]. We examined PTPN2 expression with respect to six immune cells frequently infiltrating tumors, including tumor-associated macrophages (TAMs), myeloid-derived suppressor cells (MDSCs), neutrophils, CD8^+^ T cells, regulatory T cells (Tregs), and natural killer (NK) cells. The detailed specific biomarkers of each immune cell type are listed in Additional file 7: Table S4. Canonical correlation analyses showed that glioma-derived PTPN2 expression was positively correlated with the specific marker gene expression of all six immune cell types in both the CGGA and TCGA datasets [11] (Fig. 6, *p* < 0.001), which suggests that glioma tumors with high PTPN2 expression tend to have more infiltrating immune cells than gliomas with low PTPN2 expression. These results are in agreement with the chemotaxis result in the GO analysis.

### PTPN2 predicts worse overall survival in patients with gliomas

**Fig. 7:**
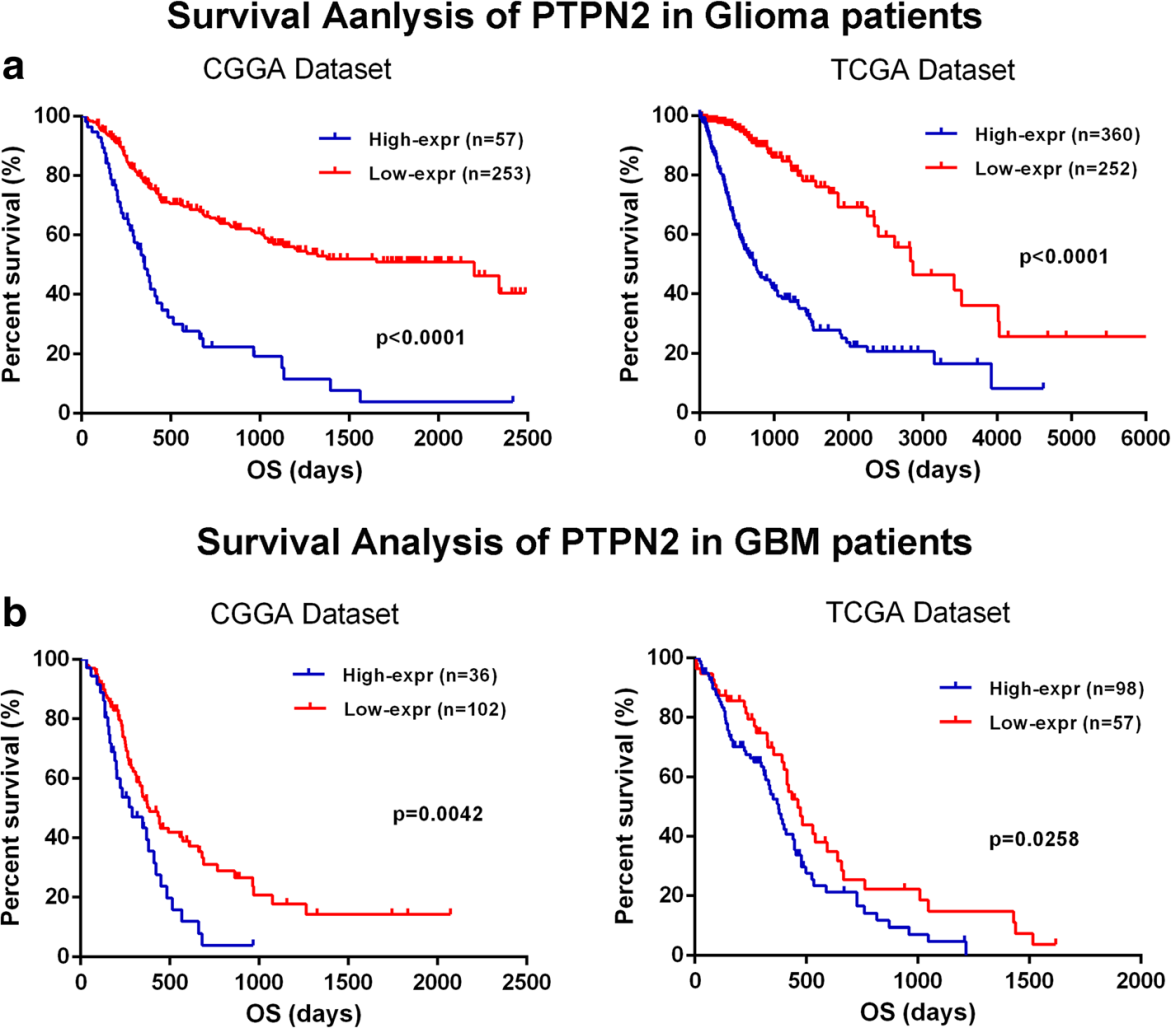
Kaplan–Meier survival analysis shows that higher PTPN2 transcript levels are associated with worse overall survival both in patients with glioma (**a**) and with glioblastoma (GBM) (**b**)

As PTPN2 plays a negative role in antitumor immunity, it might affect the survival of patients with glioma. The prognostic value of PTPN2 was evaluated in 310 and 612 patients of the CGGA and TCGA cohorts, respectively. As shown in Fig. 7a, there was a strong association between higher PTPN2 expression and shorter OS in patients with glioma. Owing to tumor heterogeneity in the different grades of glioma, the prognostic value of PTPN2 expression was also investigated in GBM. A higher PTPN2 expression also predicted worse OS in patients with GBM from both the CGGA and TCGA datasets (Fig. 7b). In the multivariate analysis aimed to prove the independent prognostic value of PTPN2, a higher PTPN2 expression was still an independent prognostic factor in patients with glioma, after adjustment of clinical factors (age, IDH mutation status, and tumor grade). The independent prognostic value was observed for both datasets (Additional file 8: Table S5).

## Discussion

ICIs are considered promising cancer therapeutics that maintain the imbalance between immune surveillance and cancer promotion. Clinical evidence proved that some ICIs, such as anti-PD-1/PD-L1 agents, are superior to conventional chemotherapy [27]. However, certain types of cancer, such as glioblastomas, are refractory to a monotherapy with ICIs [27, 28]. The special adverse events caused by ICIs cannot be overlooked either [29]. These negative results encouraged us to discover other immunotherapeutic targets and treatment strategies to treat cancer.

PTPN2 has recently been identified as a novel immunotherapy target, and deletion of this gene could increase the therapeutic efficacy of PD-1 blockade [17]. In this study, we characterized PTPN2 mRNA levels in 996 patients with glioma. We observed that PTPN2 transcript levels increased with the glioma grade and were associated with IDH wild-type gliomas and the mesenchymal subtype of GBM. Moreover, our results proved the prognostic value of PTPN2 both for patients with glioma and GBM. These results suggest a crucial role for PTPN2 in the malignant biological process of gliomas.

Next, we conducted a comprehensive analysis of the biological functions associated with PTPN2 in glioma. Similar to the expression of PD-L1, TIM3, and IDO [10, 11, 30], PTPN2 expression was closely related to the immune and inflammatory responses, as well as a higher invasion of immune cells. PTPN2 is a key negative regulator of the immune and inflammatory responses, and its single nucleotide polymorphism is linked with a high susceptibility to autoimmune diseases [31]. Moreover, loss of *Ptpn2* resulted in severe systemic inflammation and autoimmunity and increased number of immune cells in mice [32]. These results indicate that PTPN2 inhibits the immune response and is associated with autoimmunity disease. Moreover, deletion of *ptpn2* increased the sensitivity to T cell immunity in melanoma models, suggesting a novel target for immunotherapy in cancers [17]. We found a higher infiltration of antitumor immune cells (CD8^+^ T cells, TAMs, and NK cells) in gliomas with high PTPN2 expression, which is not in agreement with a previous study that reports a significant increase in the number of CD8α^+^ cells in PTPN2-deficient tumors [17]. These conflicting results need to be clarified in future studies, but it is likely to indicate that PTPN2 does not affect CD8^+^ cell infiltration in tumors. We speculate that high PTPN2 expression level is an adaptive mechanism of cancer immunosurveillance, as we found a higher number of infiltrating antitumor immune cells. Moreover, PTPN2 was shown to restrain CD8^+^ T cell responses to maintain immune tolerance in mice [33]. PTPN2 also inhibits T cell development by negatively regulating IL-7R/STAT signaling in T cell progenitors [34], and the differentiation of macrophages is negatively regulated by *Ptpn2* in mice [35]. Furthermore, a correlation between PTPN2 transcript levels and immunosuppressive cells, such as neutrophils, MDSCs, and Tregs, was observed, with neutrophils [36, 37], MDSCs [38], and Tregs [39] exhibiting strong immunosuppressive activities and contributing to poor clinical outcomes in patients with cancers. However, future studies are needed to investigate the precise interaction between PTPN2 and immunosuppressive cells. In summary, these results report PTPN2 as a negative prognostic factor in cancer. Consequently, PTPN2 could be a key element in the immunomodulation of immune cells.

The combination of various ICIs has demonstrated clinical benefits in both preclinical studies and clinical trials [7, 8, 12]. While PTPN2 blockade could be used as a monotherapy target, it could also increase the sensitivity of other ICIs in the treatment of patients with cancer. Sensitivity of the immunotherapy increased by PTPN2 loss is dependent on IFNγ signaling [17]. Moreover, IFNγ signaling is a crucial pathway in the resistance to anti-PD-1 therapy in patients with cancer [40, 41]. Thus, it can be inferred that the combination of anti-PD-1 and anti-PTPN2 therapy may be an alternative treatment method. Future research is needed to further explore our hypothesis, as we did not analyze PTPN2 protein levels with regard to clinicopathological factors, inflammatory activities, immune response, and immune cell infiltration. It is known that mRNA expression does not necessarily correlate with protein levels, as the process of transcription can be altered in cancers [42]. Thus, it will be necessary to study PTPN2 protein levels to confirm the crucial role of PTPN2 in gliomas.

## Conclusions

In conclusion, to the best of our knowledge, our study is the first to characterize PTPN2 expression levels in gliomas. Our results highlight PTPN2 as a novel immunotherapy target in glioma, which could also amplify the therapeutic efficacy when combined with other ICIs.

## Additional files

Additional file 1: **Figure S1.** PTPN2 transcript levels increase in gliomas with unmethylated MGMT promoter from the CGGA (A) and TCGA (B) datasets. (TIF 582 kb)

Additional file 2: **Table S6.** 20 genes most positive and negative significantly correlated to PTPN2 (XLSX 9 kb)

Additional file 3: **Table S1.** Immune response-related gene-Amigo 2. (XLSX 71 kb)

Additional file 4: **Table S2.** Significant immune response-related gene. (XLSX 17 kb)

Additional file 5: **Figure S2.** Heat maps displaying the gene expression levels of immune response and grade, IDH1 mutation, and MGMT promoter status from the CGGA and TCGA datasets. (TIF 15834 kb)

Additional file 6: **Table S3.** Biomarkers of each immune cell type. (XLSX 9 kb)

Additional file 7: **Table S4.** Metagenes and corresponding genes in each metagene. (XLSX 10 kb)

Additional file 8: **Table S5.** Cox analysis of prognostic factors. (XLSX 9 kb)

## Abbreviations

AUC: Area under the curve
CGGA: Chinese Glioma Genome Atlas
CNS: Central nervous system
GBM: Glioblastomas
GO: Gene ontology
GSVA: The gene set variation analysis
IDH: Isocitrate dehydrogenase
IDO1: Indoleamine 2,3 dioxygenase 1
MDSCs: Myeloid-derived suppressor cells
NK cells: Natural killer cells
OS: Overall survival
PD-L1: Programmed death-ligand 1
PTPN2: Protein tyrosine phosphatase, non-receptor type 2
TAMs: Tumor-associated macrophages
TCGA: The Cancer Genome Atlas dataset
TIM3: T cell immunoglobulin mucin-3
Tregs: Regulatory T cells
