# Supplementary figures and images for: Molecular and clinical characterization of PTPN2 expression from RNA-seq data of 996 brain gliomas
Source: J Neuroinflammation. 2018 May 15;15:145. doi: 10.1186/s12974-018-1187-4 (PMC5953404; doi:10.1186/s12974-018-1187-4)

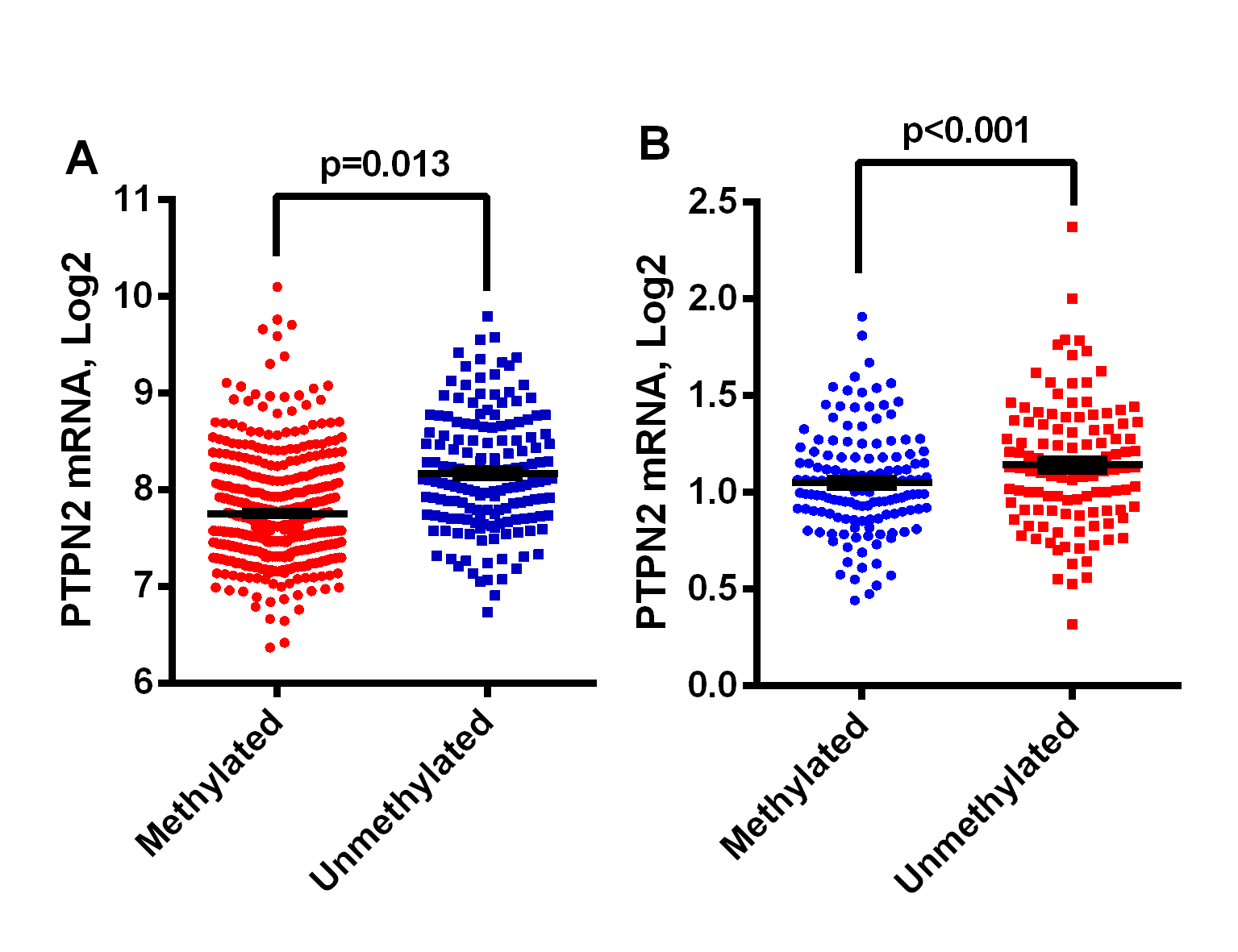

Supplement: Supplementary file 1 — Figure S1. PTPN2 transcript levels increase in gliomas with unmethylated MGMT promoter from the CGGA (A) and TCGA (B) datasets. (TIF 582 kb) [file 12974_2018_1187_MOESM1_ESM.tif]

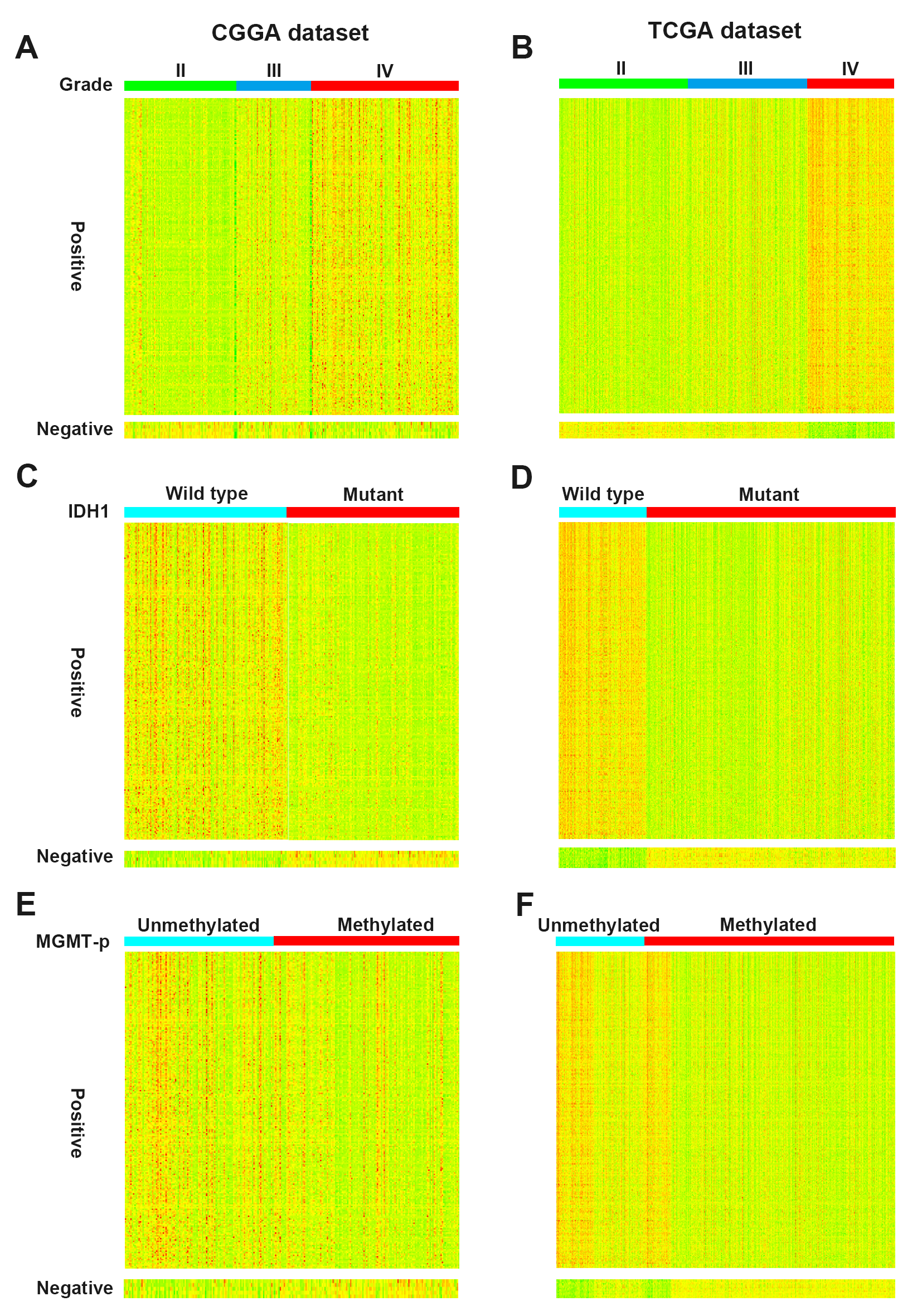

Supplement: Supplementary file 5 — Figure S2. Heat maps displaying the gene expression levels of immune response and grade, IDH1 mutation, and MGMT promoter status from the CGGA and TCGA datasets. (TIF 15834 kb) [file 12974_2018_1187_MOESM5_ESM.tif]
